# The mediating role of rumination and personal growth on pandemic depression: a longitudinal study

**DOI:** 10.1093/geroni/igab046.2692

**Published:** 2021-12-17

**Authors:** Teresa Paniagua, Virginia Fernández-Fernández, MªÁngeles Molina Martínez

**Affiliations:** 1 Universidad Europea de MAdrid, Majadahonda, Madrid, Spain; 2 Universidad Nacional de Educación a Distancia, Madrid, Madrid, Spain; 3 Universidad Francisco de Vitoria, Madrid, Madrid, Spain

## Abstract

**Introduction:**

The experience of recent months, caused by COVID-19 pandemic including strict home confinement, has required older people to implement coping strategies to combat the harmful effects of depression and associated loneliness. Method: longitudinal study, including functionally and cognitively independent people over 65, three temporal evaluation measures: WAVE1 (6 months pre-COVID-19, N=305; M=73.63; 58.9% women), WAVE2 (during home confinement; N=151; M=73.14; 59.6% women) and WAVE3 (8 months post-WAVE2; N = 85; M=72.62; 64.70% women). Bivariate correlations and a multiple hierarchical regression model are performed to explain the variance of depression in WAVE3 from rumination and growth on general life events (in WAVE1) and rumination and personal growth associated with COVID-19, both in WAVE2 and WAVE3.

**Results:**

statistically significant correlations are obtained between all the variables. The regression model explains 65.7% of the variance of depression (all steps significant). Both rumination (B=0.45;p=0.00) and growth (B=-0.40;p=0.00) on general life events (WAVE1) explain 54.5% of the depression in WAVE3. Rumination on COVID (B=.310;p≤0.01) in WAVE2 and the growth over COVID (B=-0.24;p≤0.01) in WAVE3, allow a significant explanation of 6.8 and 4.4% of the proposed model, respectively.

**Conclusions:**

it seems clear the impact that emotional regulation strategies have on life events over time. In addition, rumination is an emotional process of maladaptive coping also in the face of the pandemic. However, the growth capacity of the person is a useful tool to combat the damaging effects of negative life events. In the elderly, it seems necessary to influence and bet on positive coping strategies.

